# Viral Haemorrhagic Fevers, Perspectives in Medical Virology, Volume 11

**DOI:** 10.3201/eid1107.050609

**Published:** 2005-07

**Authors:** Peter B. Jahrling

**Affiliations:** * National Institutes of Health, Frederick, Maryland, USA

**Keywords:** Viral, Haemorrhgic, Fevers

Viral Haemorrhagic Fevers is a compact and highly readable monograph written by Collin Howard, an authoritative and veteran virologist with hands-on practical experience in this field. This volume is highly satisfying on a variety of levels. Self-contained chapters deal with each of the 4 taxonomic viral families (*Flaviviridae*, *Arenaviridae*, *Bunyaviridae*, and *Filoviridae*), which make up the category of viral hemorrhagic fever agents. The properties of each virus family are presented in terms of molecular virology and replication strategy, followed by the epidemiology, clinical presentation, and treatment options. Cross-referencing to other chapters is kept to a minimum, enhancing the readability of the text. The information is reasonably current, with the exception of the current Marburg outbreak in Angola. For each virus, the author offers his candid assessment of the available treatment options. My only quibble is that I do not share his pessimism that effective vaccines will not be developed and distributed in the near future.

The author made a conscious decision to avoid encyclopedic referencing to enhance readability. On occasion, this results in bold statements that the specialist might wish had been referenced. One example is the discussion of Whitewater Arroyo virus and its probable (but contentious) role in 3 fatal cases of hemorrhagic fever from 1999 to 2000. Another is a statement that infection of endothelial cells is a critical event in the pathogenesis of Ebola virus, which, to my knowledge, has never been adequately documented.

The text is enhanced by electron micrographs of representative agents, photographs of rodent reservoirs in their native habitat, maps of geographic distributions, and schematic representations of genome organization and replication strategies. The legend to Figure 2 in the Filovirus chapter compares Marburg and Ebola viruses, but only 1 image (which I recognize to be Marburg) is displayed.

The author states that this book was directed "primarily at healthcare workers, clinicians, and microbiologists wishing to gain a rapid overview of these widely varying agents." This book should appeal to that audience, and also to a wider audience of persons interested in the public health ramifications of these serious viral diseases in their geographic niches as well as their potential to cause disease in industrialized nations, either when imported by returning travelers or deliberately released in an act of terrorism. These aspects of viral hemorrhagic fever agents are discussed in both the introductory and concluding chapters of the book. A nice touch was the inclusion of a short annotated bibliography of books for the lay audience dealing with the history of these agents, plus a second list of specialized textbooks of more interest to the practicing virologist. Other appendices list useful Web sites and a capsule explanation of how biocontainment facilities are designed to deal with these fearsome pathogens. There is something for everyone in this book; I will keep my copy handy, next to my desk.

